# Minor taxa in human skin microbiome contribute to the personal identification

**DOI:** 10.1371/journal.pone.0199947

**Published:** 2018-07-25

**Authors:** Hikaru Watanabe, Issei Nakamura, Sayaka Mizutani, Yumiko Kurokawa, Hiroshi Mori, Ken Kurokawa, Takuji Yamada

**Affiliations:** 1 School of Life Science and Technology, Tokyo Institute of Technology, Ookayama, Meguro-ku, Japan; 2 Research Fellow of Japan Society for the Promotion of Science, Ookayama, Meguro-ku, Japan; 3 Education Academy of Computational Life Science, Tokyo Institute of Technology, Nagatsuta-cho, Midori-ku, Yokohama, Kanagawa, Japan; 4 Center for Information Biology, National Institute of Genetics, Yata, Mishima, Shizuoka, Japan; 5 PRESTO, Japan Science and Technology Agency, Saitama, Japan; Kyushu Institute of Technology, JAPAN

## Abstract

The human skin microbiome can vary over time, and inter-individual variability of the microbiome is greater than the temporal variability within an individual. The skin microbiome has become a useful tool to identify individuals, and one type of personal identification using the skin microbiome has been reported in a community of less than 20 individuals. However, identification of individuals based on the skin microbiome has shown low accuracy in communities larger than 80 individuals. Here, we developed a new approach for personal identification, which considers that minor taxa are one of the important factors for distinguishing between individuals. We originally established a human skin microbiome for 66 samples from 11 individuals over two years (33 samples each year). Our method could classify individuals with 85% accuracy beyond a one-year sampling period. Moreover, we applied our method to 837 publicly available skin microbiome samples from 89 individuals and succeeded in identifying individuals with 78% accuracy. In short, our results investigate that (i) our new personal identification method worked well with two different communities (our data: 11 individuals; public data: 89 individuals) using the skin microbiome, (ii) defining the personal skin microbiome requires samples from several time points, (iii) inclusion of minor skin taxa strongly contributes to the effectiveness of personal identification.

## Introduction

Distinct microbial communities exist all over and within the human body, and recent studies have revealed a strong correlation between the status of the bacterial composition of the gut microbiome and human disease [1,2]. The health of the human skin microbiome also has been reported to be an important factor in the development of skin diseases [3–6]. The skin microbiome of an individual varies over time [7], but such a temporal variability is less than inter-individual variability for more than 2.5 years [8,9]. Thus, the human skin microbiome has the potential to identify individuals. In a pioneering study, samples of residual human-skin microbes from computer keyboards facilitated the identification of three individuals [10]. Notably, particular microbes in the human skin have often been considered for use in forensic science [11]. For example, recent studies have emphasized that single-nucleotide polymorphism (SNP) or short-tandem repeat (STR) variations in the human skin microbes are beneficial for determining individuals in populations of >10 individuals, and the variation is stable over a prolonged period (>2.5 years) [9,12,13]. Despite being high-accuracy methods for personal identification, these methods used only major bacterial taxa. Previously, not only particular bacterial taxa but also the human microbiome composition could be used for personal identification to some extent. It is imperative to elucidate how the microbial composition defines the personal skin microbiome and what taxa are acquired/lost or sustained by the personal skin microbiome. A recent attempt for identifying 50 individuals using the human stool microbiome precisely predicted the owners of samples with >80% accuracy using the stool samples collected 30–300 days prior [14]. However, with the skin microbiome (e.g., antecubital fossa, retroauricular crease, and anterior nares), this method was successful for only <30% of individuals in a population of 61–85 individuals, although their method had a low rate of false-positives. This finding signifies that human skin microbial composition cannot be used for the personal identification for larger numbers of individuals.

In this study, we aimed to develop a highly accurate personal identification method using the human skin microbiome. Briefly, the objectives of this study were as follows: (a) the development of a novel personal identification method using human skin microbiome compositions and (b) the determination of what kind of taxa contribute toward highly accurate personal identification. In this study, we implemented our novel approach for two sets of cohort data (our data: 11 individuals; public data: 89 individuals). Contrary to previous research [14], we selected the forehead microbiome as a skin microbiome model because we anticipated that this part of the body is less likely to come in contact with clothing or other individuals and is usually exposed; hence, the forehead typically experiences the same environmental conditions year-round. In fact, the microbiome of the glabella, which is adjacent to the forehead, is reportedly relatively stable compared with microbiomes at other skin sites [15]. According to our personal identification approach, we considered minor taxa of the skin microbiome to be an important factor for distinguishing individuals. We also created an individual human skin microbiome dataset from multiple time-point samples; preparing such samples was found to be effective for personal identification. Our personal identification using the human skin microbiome successfully identified individuals even in large amount of individual community. Moreover, using our method, we found that high accuracy of personal identification using the human skin microbiome requires minor taxa.

## Materials and methods

### Subject recruitment and sampling

**Fig 1:**
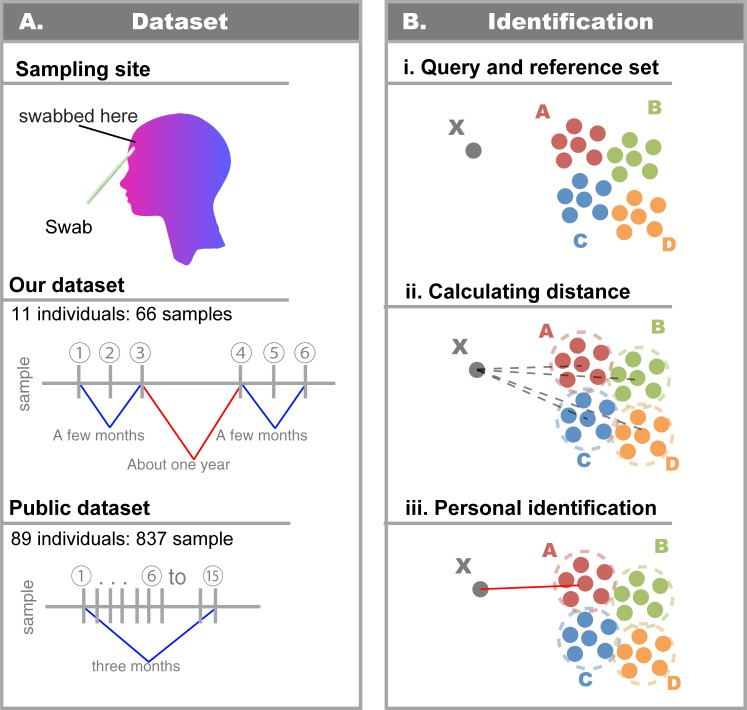
Overview of samples and personal identification. A) The collection of samples, which were taken from the forehead of 11 individuals at three time points in each of two years. Likewise, public data were sampled from forehead at 6 to 15 time points over 3 months (ERP00512) [7]. For each sample, we analyzed the microbial community composition using 16S rDNA amplicon sequences. B) The personal identification flow is described. We then developed a classifier to identify individuals based on microbial community compositions. i) X is the query, and known samples are defined as references. ii) The mean distance between query and reference is calculated. iii) We assign query X to a reference individual in the according to the distance, and assess the individual is true owner of sample X or not.

This research was approved by the ethics committee of Tokyo Institute of Technology (approval number: 2013003). We recruited healthy volunteers (10 males, 1 female, 20 to 30 years old) from Tokyo Institute of Technology (S1 Table and Fig 1A). All subjects signed a written informed consent form in accordance with the sampling procedure approved by the Institutional Ethics Committee. All individuals reported whether they had used an antibiotic in the preceding ~3 months. In this regard, individual K used antibiotics between sampling points 5 and 6, but no other subject reported antibiotic use near the time of any of the sampling points.

All subjects were asked to abstain from washing their forehead for two hours before sampling. The samples were collected from a 4 cm^2^ area at the center of the forehead by dry swabbing 50 times back and forth with a sterile swab. Samples were collected at three time points in the first year and three time points in the second year. The samples were stored in dry 1.5 ml tubes at −20°C. At approximately the same time points, we measured three skin parameters, namely moisture, pH, and sebum, near the sampling area on the forehead. These measurements were made with instrumentation from Courage and Khazaka Electronic, GmbH: moisture was measured using a Corneometer (Model CM820), pH was measured using a pH meter, and the sebum was measured using a Sebumeter (Model SM810 PC). Median values were calculated from three replicate measurements.

### DNA extraction and sequencing

Genomic DNA was extracted from the skin swabs using the Purelink Genomic DNA Mini kit (Thermo Fisher Scientific). To assess the microbial taxonomic composition of each sample, the 16S rDNA were PCR amplified using the universal primer set 27F (5′-AGAGTTTGATCCTGGCTCAG-3′) and 338R (5′-TGCTGCCTCCCGTAGGAGT-3′) and Ex Taq DNA polymerase, Hot-Start Version (TaKaRa Bio). PCR was carried out in 50 μl reactions containing 0.2 μM forward and reverse primers, 20 μl template DNA, 5 μl 10× Ex Taq buffer (TaKaRa Ex Taq, Hot-Start Version), and 4 μl dNTP mix (TaKaRa Ex Taq, Hot-Start Version). The PCR program consisted of 95°C for 5 min followed by 31 cycles of 95°C for 30 sec, 53°C for 30 sec, and 72°C for 30 sec, with final extension at 72°C for 3 min. The PCR products were individually concentrated and purified using a 2% E-Gel SizeSelect agarose gel (Thermo Fisher Scientific) and quantified using the Quant-iT dsDNA HS Assay kit (Thermo Fisher Scientific) and the High Sensitivity DNA kit (Agilent Technologies). Sequencing was carried out with the Ion PGM Sequencing 400 kit (Thermo Fisher Scientific). We used sterile swabs as negative control samples and performed the same method—from sampling to PCR—with the negative control swabs. We did not observe PCR products after electrophoresis of the reactions through a 2% agarose gel, indicating that there was no contamination in negative control.

### Sequence analysis

In the analysis of our original dataset, to obtain high-quality 16S rDNA amplicon sequences, we removed reads of <200 nt, trimmed the low-quality regions within the 5´-end and 3´-end (quality score ≤ 17), and removed reads with an average quality score of <25 using Trimmomatic version 0.33 with the parameter ‘LEADING:17 TRAILING:17 AVGQUAL:25 MINLEN:200’ [16], and we removed primer regions containing three or more mismatches using TagCleaner version 0.12 with the parameter ‘-mm5 3 -mm3 3 -nomatch 3’ [17]. After removing the primer region, we performed ‘-sortbylength’ command of USEARCH with parameter ‘-minseqlength 200’ to remove reads of <200 nt from removing primer section [18]. We performed clustering method of sequences for resolving the sequence error from Ion PGM sequences. We combined two clustering methods to ensure accurate clustering: UCLUST and MCL. To compress the large amount of sequences for operational taxonomic unit (OTU) assignment, we performed high-identity clustering using UCLUST version 7.0.1001 with a minimum coverage of 90% and minimum identity of 98% [18]. We then removed the chimeric sequences using UCHIME version 7.0.1001 in de novo mode and reference mode with GOLD DB (http://drive5.com/uchime/gold.fa) [19]. The UCLUST algorithm alone was not able to correctly cluster the data [20]; therefore, to confirm the OTUs, the 74,955 UCLUST OTU sequences were pairwise aligned in an all-against-all BLASTN search with the parameter ‘-e 1e-8 -b 74,955 -v 1’ and clustered with an identity threshold of ≥97% and coverage ≥80% using MCL version 14–137 with default parameters [21,22]. Representative OTU sequences were taxonomically assigned to species registered in the All-Species Living Tree project (LTP) version 128 [23], which is a 16S rDNA sequence database of Archaea and Bacteria type strains, using BLASTN searches with identity ≥97% and alignment coverage ≥80%. All species hit by BLASTN parameter are listed (S2 Table).

In the analysis of the public dataset (ERP00512) [7] (Fig 1A), we downloaded fastq file of ERP00512 from European Nucleotide Archive (ENA). We selected 89 individuals, who have more than six time points of forehead microbiome samples, and performed 16S rDNA analysis to their samples. To obtain high-quality 16S rDNA amplicon sequences, we removed reads of <75 nt, trimmed the low-quality regions within the 5´-end and 3´-end (quality score ≤ 17), and removed reads with an average quality score of <25 using Trimmomatic with the parameter ‘LEADING:17 TRAILING:17 AVGQUAL:25 MINLEN:75’ [16]. To create the 97% OTU table, we utilized UPARSE which is installed in USEARCH version 10.0.240 with default parameters [24], which is efficient with respect to computational requirements and provides rapid clustering because of our need for time-consuming computations for our 16S rDNA amplicon analysis (such as all-against-all BLAST).

### Statistical analysis

To assess the α-diversity of OTUs of each sample, the Shannon diversity index was calculated and compared among samples using VEGAN package version 2.4–2 of R version 3.0.2 (or more recent version 3.3.2) [25,26]

To assess the variability of each of intra- and inter-individual taxonomic composition, we calculated the Canberra distance between samples using R. Canberra distance normalizes the absolute difference in abundance of each taxon, thereby allowing comparison of minor taxa. A shorter Canberra distance indicates greater similarity. For the taxonomic composition of samples *X* and *Y*, (*X*_*1*_, …., *X*_*n*_) and (*Y*_*1*_, …., *Y*_*n*_), the Canberra distance is Eq (1):

$$D_{canberra}(X,Y)=\sum_{i=1}^{n}\frac{\left|X_{i}-Y_{i}\right|}{\left|X_{i}|+|Y_{i}\right|}$$ (1)

Differences in Canberra distances derived for inter-individual sample pairs and intra-individual sample pairs from the same year and across years were statistically assessed with the Wilcoxon rank-sum test using R software.

### Personal identification method

We developed the following personal identification method based on the human skin taxonomic composition (Fig 1B). First, reference samples (known individuals) and query samples (unknown individuals) were defined; then, the Canberra distance between a query sample and reference samples was calculated using R software. The mean Canberra distance between a query sample and each individual’s reference samples, defined as *D*_*individual*_(*q*, *r*_*individual*_), was compared using Eq (2):

$$D_{individual}(q,r_{individual})=\frac{\sum_{i=1}^{n}D_{canberra}(q,r_{i})}{n}$$ (2),

where *q* is the taxonomic composition of the query sample, *r*_*i*_ is the taxonomic composition of the samples of individual *i*, *n* is the number of samples for each individual, and *D*_*canberra*_(*q*,*r*_*i*_) is the Canberra distance between *q* and *r*_*i*_. Finally, the reference individual with the minimum *D*_*individual*_(*q*, *r*_*individual*_) value against the query was defined as the owner of the query sample.

To validate our personal identification method, we calculated the true positive (TP) rate using Eq (3):

$$Accuracy\;of\;personal\;identification=\frac{N_{TP}}{N_{TP}+N_{FN}}$$ (3),

where *N*_*TP*_ is the number of true positives (query data accurately assigned to the original individual), and *N*_*FN*_ is the number of false negatives (FN). To avoid the situation that the query sample is contained within the reference samples in the training data, we extracted the query samples from the training dataset during the leave-one-out validation. An open source R-script on personal identification using the human skin microbiome is available on the Github repository (https://github.com/yamada-lab/human_skin_personal_identification).

In addition, we extended our method to the case that lacked TP in the reference dataset (S4A Fig). Accordingly, we set a threshold of the Canberra distance to determine TN or FP. In the validation of the TP, we created a situation that the owner of a query is in the reference. If the query sample was the closest to the true owner’s and the Canberra distance was less than the threshold, we considered it as TP, whereas other cases were judged as FN or FP. In the validation of the TN, we created a situation that the owner of a query is not in the reference dataset. If the query sample was the closest to someone’s and the Canberra distance was less than the threshold, we judged it as FP. Another case was judged as TN. We performed these tests using the leave-one-out method with the public dataset [7] and each threshold of the Canberra distance (14,000–18,000 in 100 increments).

### Parameters for personal identification method

To assess the influence of the number of reference samples (1 to 5 samples) per individual on the accuracy of the personal identification method, we used public dataset for a large number of samples from the same individuals collected at several time points [7]. The reference data were randomly selected from each individual’s samples, and query data were randomly selected from one of the remaining samples from each individual. To validate the prediction accuracy according to the number of reference samples, we carried out 100 tests for each randomly selected number (1 to 5 samples) of reference samples and performed our personal identification method.

To assess the time effect of sample collection, we used our original data to calculate the accuracy query–reference sample pairs taken within the same year and in consecutive years. We carried out 100 tests for each pair taken within the same year and in consecutive years. First, we randomly selected one reference sample per individual. Second, one query sample randomly selected from the samples that remained in the first step were used to carry out our personal identification method and calculate the accuracy.

Low abundant taxa are often removed by a particular cut-off value to avoid the effect of a sequence error or contamination. We transformed relative abundance values of microbial OTUs, which were lower than each cut-off value (1e–01, 1e–02, 1e–03, 1e–04, 1e–05, 1e–06, and 0) to zero to evaluate the effect of a cut-off value for bacterial relative abundances on the accuracy of our personal identification method and performed our personal identification method using the transformed OTU dataset.

To assess the effect of the number of reads for bacterial relative abundance on personal identification accuracy, we randomly sampled reads from each microbial OTU table using Rarefy function of GUniFrac [27] version 1.0 of R package for each read depth (100, 1000, 10000, 20000), and we performed our personal identification method using the transformed OTU dataset. The evaluation was performed 10 times.

### Detection of personal OTU in the skin microbiome of individuals

We defined “personal OTU”, which are considered to contribute toward making a personality for the skin microbiome. We detected the presence of OTUs in all time-points from the same individual to find the personal OTU. Optionally, the median abundance of these OTUs in six samples from the same individual must be larger than two times (log_2_ fold-change ≥1) of the median abundance of these OTU from other individuals. In addition, we estimated the origin of these OTUs from isolation information of the type strains assigned to the OTU. Based on the isolation information of the type strain, we manually categorized all origins of the OTU into nine categories (human skin, human-related, air, soil, plant, water, other, unknown, and unclassified; S3 Table). Bacterial species categorized as “unknown” did not provide any information about the site of isolation. Moreover, the “unclassified” bacteria could not be assigned to any type strain or multiple hit to type strains.

### Correlation between the relative abundance of OTU0001 and skin physiological parameters

To identify relationships between specific skin microbial taxa and skin characteristics, we calculated Spearman’s correlation coefficients between three skin physiological parameters (moisture, pH, and sebum) and the relative abundance of the most abundant OTU0001 for each individual. We used only OTU0001 in the physiological analysis of our dataset because other OTUs, except for OTU0001, were tremendously less than OTU0001. Thus, other OTU relative abundance might be affected by the abundance variance of OTU0001. Hence, we selected only OTU0001.

## Results

### Taxonomic composition of human skin microbiome

We obtained 66 forehead microbiome samples from 11 individuals at six different time points over two years (Fig 1A and S1 Table). Our sequencing of the 16S rDNA region from the samples yielded 7,005,342 high-quality reads. To reveal the taxonomic composition of the skin microbiome at the species level, we computed 97% identity OTUs after sequencing the 16S rDNA amplicon that could be clustered into 4,155 OTUs (S2 Table), of which 659 OTUs could be classified into type strains, 492 OTUs were multiple hits to type strains, and 3,004 OTUs were unclassified at any type strain. The most notable result was that four dominant OTUs, namely OTU0001 (*Propionibacterium acnes*), OTU0002 (*Staphylococcus*), OTU0003 (*Corynebacterium tuberculostericum / accolens*), and OTU0004 (*Staphylococcus*), were observed in all samples.

**Fig 2:**
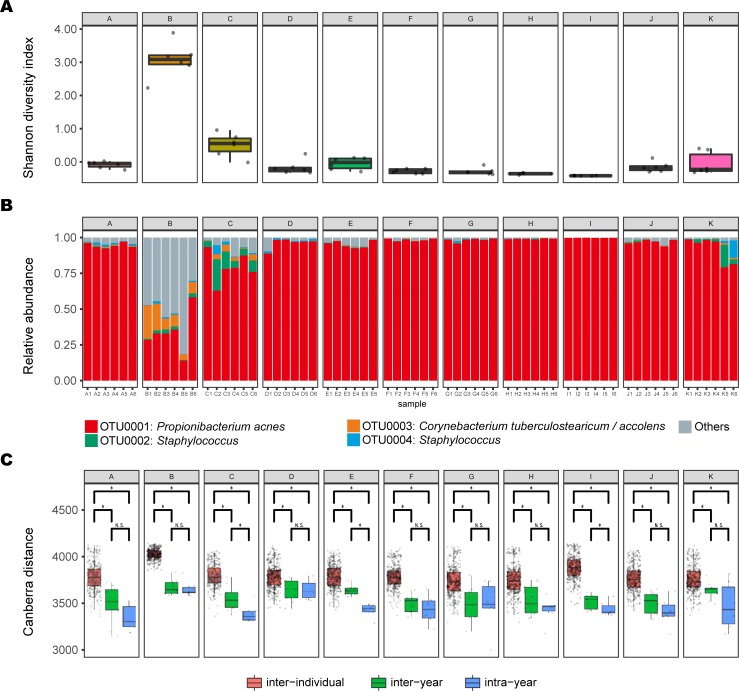
Microbial communities of 11 individuals. A) Shannon diversity index values for the six time-point samples of individuals A to K. B) Relative abundance of OTUs in samples from the six time points for individuals A to K. For each individual, the first three columns represent first-year samples, and the remaining three columns represent the second-year samples. C) Each point represents the Canberra distance between a pair of samples grouped by three comparison schemes. *P ≤ 0.05 (Wilcoxon rank-sum test). N.S., not significant.

We calculated the Shannon diversity index for each sample using OTU relative abundance. Most of the individuals had relatively low Shannon diversity at all time points, although two individuals had relatively high values (Fig 2A, individuals 2B and 2C). This tendency was also observed with respect to the distribution of OTU abundance (Fig 2B); specifically, the abundance of OTU0001 for each of individuals B and C was relatively low compared with other individuals. OTU0001, representing *P*. *acnes*, was the dominant species (accounting for ~90% of the microbial composition) in almost all samples. To assess the intra-individual stability of the skin microbiome, we measured differences in taxonomic composition over the six sampling points and compared them against other individuals. The Canberra distances between individuals were significantly greater than those within individuals (Wilcoxon rank-sum test, *P* ≤ 0.05). Hence, the intra-individual distance of the skin microbial community through one year was smaller than the inter-individual distances (Fig 2C).

In publicly available data (ERP00512) [7](Fig 1A), we obtained 46,701,064 reads for the 16S rDNA amplicon data from 837 forehead samples from 89 individuals.

### Personal identification using the human skin microbiome

We assessed the accuracy of our new personal identification approach with skin microbiome profiles using our original dataset (66 samples from 11 individuals) and leave-one-out validation (refer Materials and Methods) and evaluated a personal identification accuracy of 95% (63/66), implying that 63 samples were TP and three were FP (Table 1). Moreover, we tested the accuracy of the method when the reference and query samples were acquired in different years. Using three reference samples from the first year and three query samples from the second year, we found the accuracy to be 85% (28/33).

**Table 1:** Results obtained using the personal identification method.

Data	Query	Reference	Number of individuals	Number of samples	Accuracy
Our data	all samples	all samples	11	66	0.95
Our data	first year	second year	11	33	0.85
Our data	second year	first year	11	33	0.85
Public data	all samples	all samples	89	837	0.78

**Fig 3:**
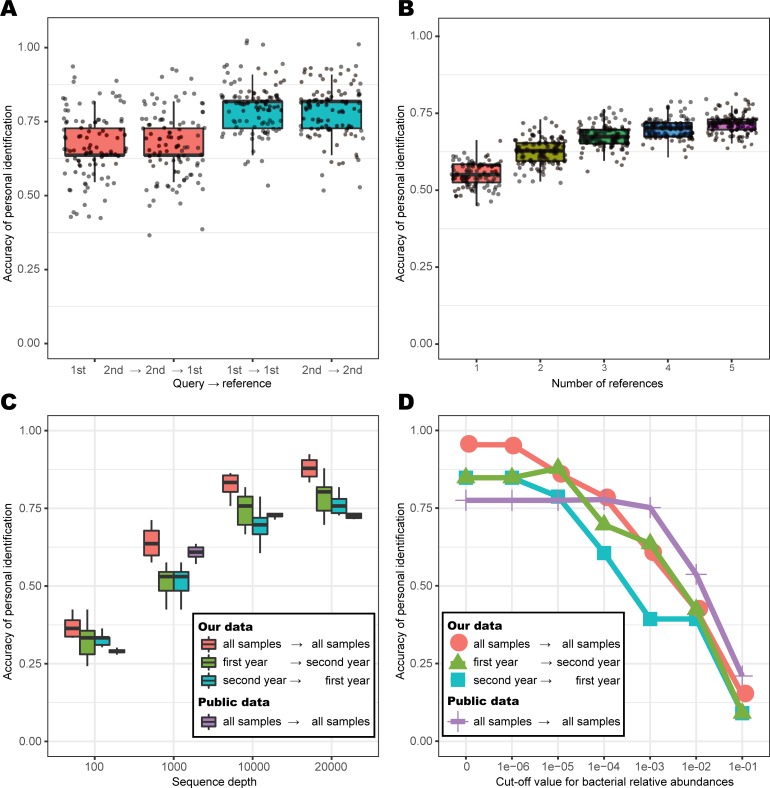
Boxplot for the accuracy of assessing the effects of each factor. A) Accuracies derived from reference samples from within the same year as the query and from different years. B) Accuracy of identifying individuals with consideration of different numbers of reference samples. *P ≤ 0.05 (Wilcoxon rank-sum test). C) Effect of the cut-off value for bacterial relative abundance on personal identification accuracy. D) Effect of the number of reads for bacterial relative abundance on personal identification accuracy.

In addition, to determine the appropriate time points for the reference samples, we calculated and compared the accuracy of personal identification between reference samples collected within the same year and those collected in different years. The results indicated that the use of samples from within the same year for reference is slightly more accurate than using samples from different years (Fig 3A). Furthermore, we evaluated the personal identification approach using a public dataset (89 individuals) and calculated a personal identification accuracy of 78% (663/837) (Table 1). For this prediction, we first constructed a personal reference microbiome for each individual. To estimate the appropriate number of samples for use in constructing the reference, we evaluated the relationship between the accuracy of personal identification and the number (1 to 5 sample) of reference samples per individual (Fig 3B). The results indicated that the accuracy of our personal identification method dramatically increased when four or five reference samples were used. Furthermore, we found that the accuracy of personal identification increased with the depth of sequencing (Fig 3C). Likewise, we examined whether a cut-off value for bacterial abundance had any effect on personal identification; we found that the accuracy increased when we used low cut-off values (Fig 3D).

We compared the Canberra distance and other distance methods (Bray–Curtis and Jaccard) to elucidate that the Canberra distance is efficiently performed in our personal identification. Consequently, we determined that the accuracy was lower than the Canberra distance method (S3 Fig).

In contrast, our method cannot work when the query individual is absent in the reference samples. We set a threshold of the Canberra distance (S4A Fig) to expand our personal identification method; this concept can distinguish between TN and FN in query samples. Consequently, it is possible to identify individuals when both TN and TP ratio is about 60%, and the threshold *d* is approximately 16,000. In this study, we designed this method to obtain higher TP or TN ratio by changing the threshold (S4B Fig).

### Correlation analysis between the relative abundance of skin microbial communities in OTU0001 and skin moisture, pH, and sebum

We considered that the skin microbiome might be affected by various aspects of human skin physiology, e.g., moisture, pH, and sebum. We determined that the OTU0001 (*P*. *acnes*) abundance was partly associated with these skin physiological parameters. Interestingly, the Spearman’s correlation coefficients differed among individuals (S2 Fig). For example, the correlation values for the relative abundance of OTU0001 and skin moisture of individuals A, G, and K were greater than 0.5. In contrast, the correlation values for OTU0001 and skin moisture of individuals B and E were less than −0.5. With respect to sebum content, the relative abundance of OTU0001 and sebum were negatively correlated for almost all individuals. This was particularly true for individuals C and D, whereas it was not necessarily the case for most other individuals. For individual C, we detected only significant correlations (P ≤ 0.05) for pH and sebum, according to Spearman’s test.

## Discussion

In this study, we collected 66 samples of the human skin microbiome from 11 individuals over 2 years. In addition, we downloaded public data for the forehead microbiome of 89 individuals to assess the accuracy of the personal identification method using other skin microbiome datasets. The most abundant bacterial genus in our 66 samples was *Propionibacterium*. Several studies have already reported *Propionibacterium*, *Corynebacterium*, and *Staphylococcus* as the primary bacterial species on the human skin [28–32]. Likewise, *Propionibacterium*, *Corynebacterium*, and *Staphylococcus* were observed in our collected forehead microbiomes obtained from 11 individuals (comprising almost all Japanese males). In addition, our samples had abundant *P*. *acnes*; however, the relative abundance of *P*. *acnes* in the human skin microbiome has been estimated to be low based on a PCR primer set [33]. Besides, the abundance of *P*. *acnes* in the public data was estimated relatively low, which could be attributed to the primer set (515F-806R). In fact, the metagenomics analysis, which has no primer bias, reported that *P*. *acnes* was dominant in the human skin microbiome, which is why we did not merge our original data with this public data [9].

The usage of our data to investigate the intra- and inter-individual differences in the taxonomic composition revealed that the taxonomic composition of the skin microbiome was mostly stable over a short period (i.e., up to a few months), but fluctuated slightly over extended periods (i.e., >1 year), suggesting that the intra-individual taxonomic composition of the human skin microbial community was relatively stable, that is, the skin microbiome was sufficient to identify an individual. This result is consistent with a prior metagenomic study [9]. Thus, we propose a new personal identification approach using skin microbiome profiles (see Methods, Personal identification approach; Fig 1 and S1 Fig). Although our personal identification method was assessed using a large number of subjects, the estimation of the accuracy for a larger cohort, such as >1 million, remains challenging because of the consequent increment of similar microbiome patterns.

Some highlights of our personal identification method are as follows: (1) low abundant taxa were not removed by the sequence abundance cut-off, and (2) some time-point samples were used as reference dataset of owners. Our method was conducted without a cut-off value for the relative abundance of taxa, implying that even minor taxa could be used to identify individuals. Typically, minor microbiome taxa were considered as contaminants and, thus, not included in the analysis [34–36]. However, we assessed whether a cut-off value for the bacterial abundance exerted any impact on the personal identification and determined that the accuracy increased upon using low cut-off values (Fig 3D). Likewise, we established that the accuracy of the personal identification increased with the sequencing depth (Fig 3C). These findings suggest that the minor taxa of the skin microbiome can contribute to the identification of individuals significantly. Thus, cut-off values should be set only after careful consideration of the relative abundance of the minor taxa, although the minor taxa were hardly observed owing to stochastic issues. Furthermore, this study illustrates that a snapshot of the skin microbiome is inadequate to define the personal microbiome because our data revealed temporal changes in the taxonomic composition of the microbiome. Hence, multiple samples from the same individual are required to define a personal skin microbiome (Fig 3B). Apparently, it is difficult for a forensic study to obtain multiple samples. If we cannot obtain multiple samples within the required time interval, we recommend deep sequencing of one sample and avoiding the inadvertent threshold of cut-off values (Fig 3C and 3D). Furthermore, the method might be effective when skin microbiome samples are sampled multiple times by slightly shifting the sampling site.

The expanded version of our method can categorize query samples into TN and TP with around 60% accuracy. Notably, the TP ratio is less than the original version of our method. However, the concept of the expanded version is crucial to prevent the misclassification as FP.

Previous studies reported that taxa are shared between the human skin microbiome and microbiomes of the environment [37–39]. In this study, we also obtained the skin microbiome that contained some forms of taxa found not only on the human skin but also other environments (e.g., soil, human gut, and plant). In addition, we even isolated taxa defined as “personal OTU” from another environment (refer Materials and Methods). Remarkable, these “personal OTU” are also found in other environments, implying that the skin microbial community reflects differences in the personal behavior or living environment (S3 Table). For example, some personal OTUs were found to have originated from water-associated environments. A recent study reported that the time an individual spends outdoors correlates positively with the number of these OTUs (termed “indicator OTUs” in their study) [40]. This observation encourages us to conduct another study to reveal taxa which is discriminant for personal identification.

In addition, this study reveals that the correlation strength between the relative abundance of OTU0001 (which had the largest relative abundance in our dataset) and physiological parameters varied among individuals (S2 Fig). In other words, correlations were highly positive for a proportion of individuals but profoundly negative for others. Thus, the correlations between the relative abundance of OTU0001 and human skin physiological parameters are unique to each individual. Reportedly, the prevalence of *Propionibacterium* increases with an increase in the cheek sebum [41]. However, this might be a consequence of the fact that the gene content of bacterial species differs among individuals. In fact, previous studies have reported that the subtypes of *P*. *acnes* on the human skin differ among individuals, and their gene contents vary from each other, thereby considering it as a factor of acne vulgaris [42,43]. However, 16S rDNA analysis was difficult to classify *Propionibacterium* to strain levels. Thus, we need another approach to get information of *Propionibacterium* strain to analyze the correlation between the microbial relative abundance with a resolution of strain/species and physiological data of the human skin.

Although this study illustrated high accuracy even for a 1-year period and across gender with a relatively large number of individuals, the results are an integration of our data and public data, implying that large public datasets are lacking and, thus, are inadequate with the time, gender, and number of individuals. For practical applications, an extensive dataset of sampled skin microbiome is required to be tested with accuracy. Moreover, our personal identification method requires re-clustering when new samples are obtained, which are either query or references; however, it remains challenging for the practical usage. Hence, we considered to map that reads directly to the 16S rDNA database to classify reads to a particular taxon. At present, some 16S rDNA databases (e.g., SILVA ribosomal RNA gene database, Greengenes Database, and Ribosomal Database Project) are publicly available to classify the 16S rDNA sequence to the taxonomy of bacteria and archaea [44–46]. Using these databases, we would not need re-clustering. However, this method cannot annotate reads that are not mapped to the present taxa in the database.

Overall, this study suggests that any microbial analysis of the human skin should consider the temporal dynamics of the skin microbiome, minor taxa. Especially, considering minor taxa is crucial for our personal identification method. However, our personal identification method should be improved for practical applications because it has not entirely classified samples to the true owner. Besides, our method requires to be tested further to verify its accuracy or should be combined with other personal identification methods for the practical usage.

Although our study comprises a small cohort, we could observe some OTUs, which were more represented in all time-points from an individual; these OTUs might be from another environment. The skin microbiome composition might provide us information as a log for individual visitation of the environment. The next objective for human skin microbiome studies will be to elucidate the environmental distribution of OTU, which distinguishes between individuals’ skin microbiome and their association with human lifestyle.

## Supporting information

S1 Fig Overview scheme for the personal identification pipeline. (PDF)

S2 Fig Correlation analysis of OTU0001 relative abundance and human skin physiological parameters. (PDF)

S3 Fig Comparison with the accuracy of Canberra, Bray–Curtis, and Jaccard distance for personal identification. (PDF)

S4 Fig The personal identification validation method for identifying true negative and true positive with the threshold of distances using public data (ERP00512) [7]. The ratio of TN and TP using our personal identification method by the leave-one-out method.
(PDF)

S1 Table Overview of the skin physiological data of the subjects. (XLS)

S2 Table OTU matrix for each individual (annotated read numbers). (XLS)

S3 Table List of OTUs which were present in all time-points from the only one individual. (XLS)
