# Supplementary material for: Minor taxa in human skin microbiome contribute to the personal identification
Source: PLoS One. 2018 Jul 25;13(7):e0199947. doi: 10.1371/journal.pone.0199947 (PMC6059399; doi:10.1371/journal.pone.0199947)
Supplement: S1 Fig — (PDF) [file pone.0199947.s001.pdf]

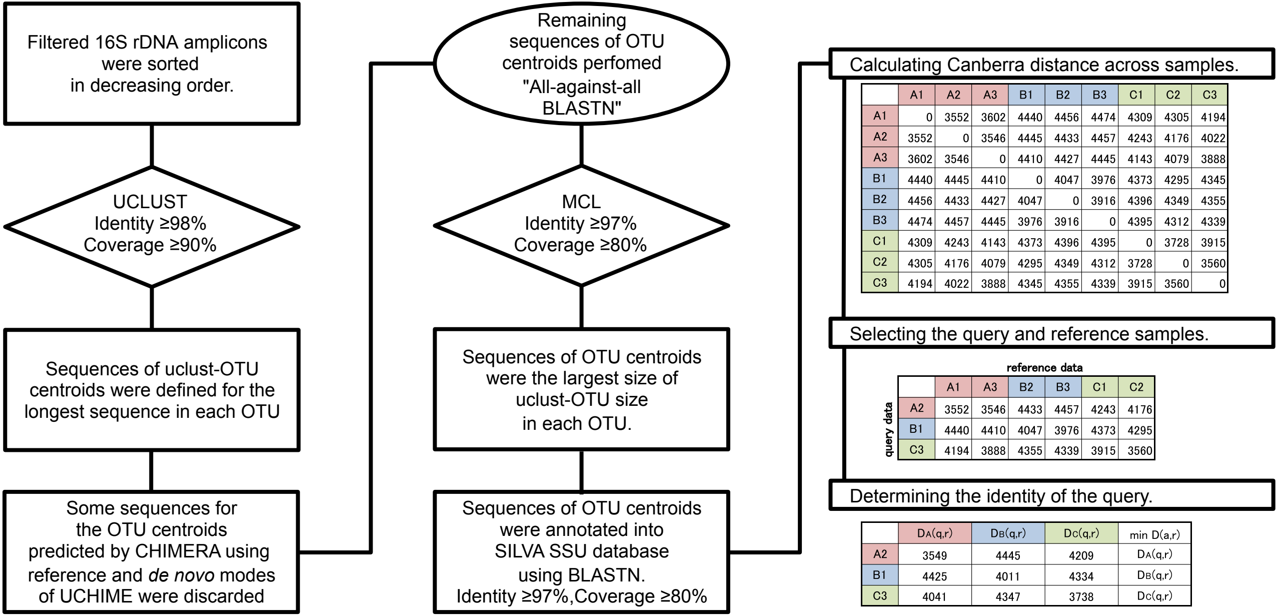

	A1	A2	A3	B1	B2	B3	C1	C2	C3
A1	0	3552	3602	4440	4456	4474	4309	4305	4194
A2	3552	0	3546	4445	4433	4457	4243	4176	4022
A3	3602	3546	0	4410	4427	4445	4143	4079	3888
B1	4440	4445	4410	0	4047	3976	4373	4295	4345
B2	4456	4433	4427	4047	0	3916	4396	4349	4355
B3	4474	4457	4445	3976	3916	0	4395	4312	4339
C1	4309	4243	4143	4373	4396	4395	0	3728	3915
C2	4305	4176	4079	4295	4349	4312	3728	0	3560
C3	4194	4022	3888	4345	4355	4339	3915	3560	0

reference data

query data	A1	A3	B2	B3	C1	C2
A2	3552	3546	4433	4457	4243	4176
B1	4440	4410	4047	3976	4373	4295
C3	4194	3888	4355	4339	3915	3560

	$D_A(q,r)$	$D_B(q,r)$	$D_C(q,r)$	min $D(a,r)$
A2	3549	4445	4209	$D_A(q,r)$
B1	4425	4011	4334	$D_B(q,r)$
C3	4041	4347	3738	$D_C(q,r)$
