# Supplementary material for: Minor taxa in human skin microbiome contribute to the personal identification
Source: PLoS One. 2018 Jul 25;13(7):e0199947. doi: 10.1371/journal.pone.0199947 (PMC6059399; doi:10.1371/journal.pone.0199947)
Supplement: S2 Fig — (PDF) [file pone.0199947.s002.pdf]

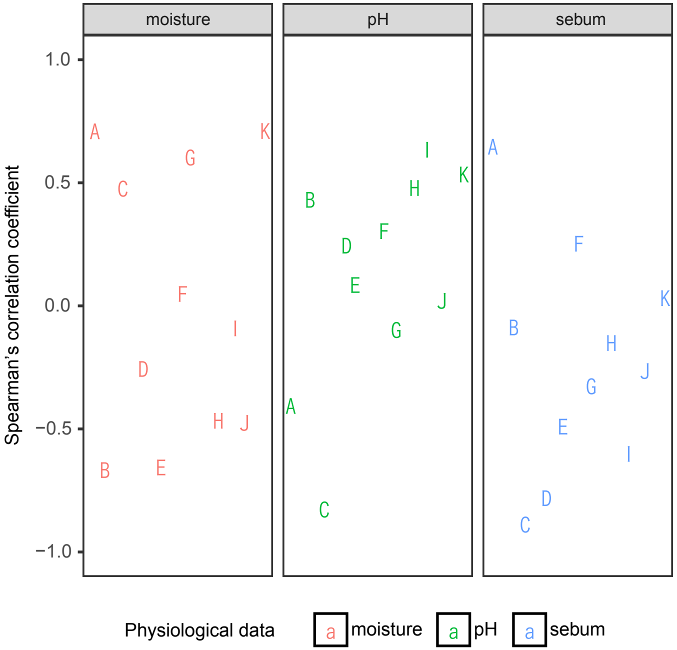
moisture
pH
sebum
Spearman's correlation coefficient
1.0
0.5
0.0
−0.5
−1.0
A
B
C
D
E
F
G
H
I
J
K
Physiological data
a moisture
a pH
a sebum
