# Supplementary figures and images for: Minor taxa in human skin microbiome contribute to the personal identification
Source: PLoS One. 2018 Jul 25;13(7):e0199947. doi: 10.1371/journal.pone.0199947 (PMC6059399; doi:10.1371/journal.pone.0199947)

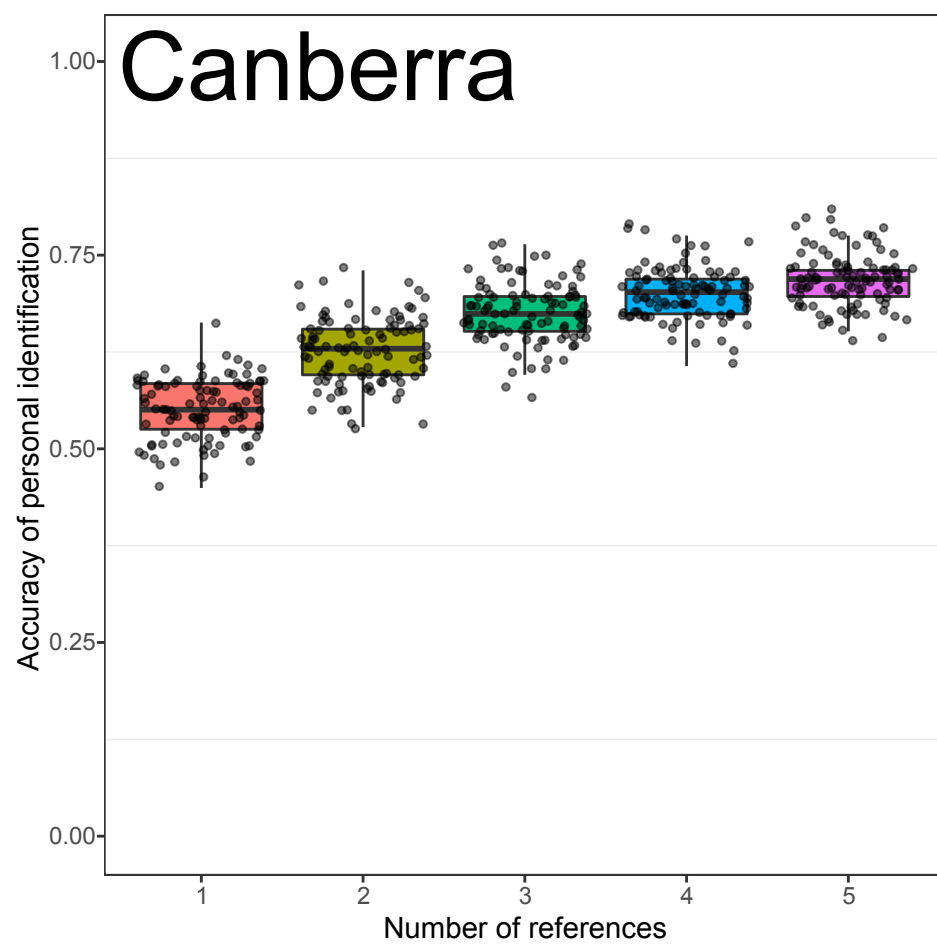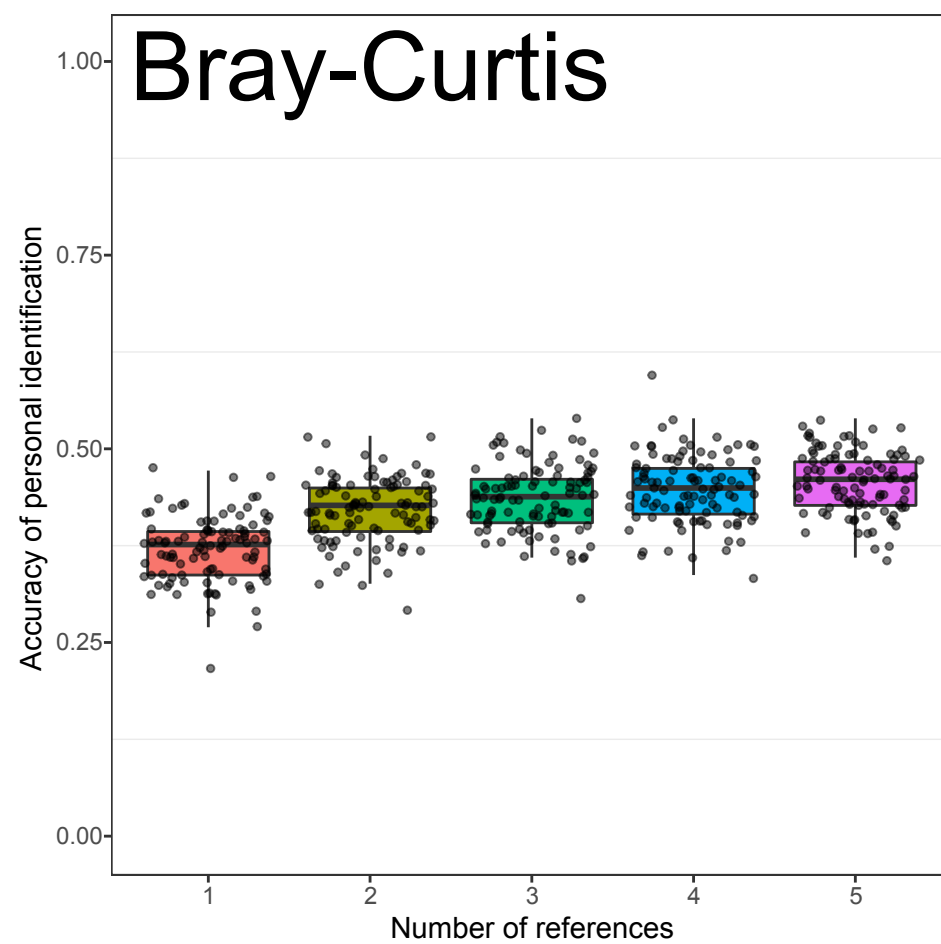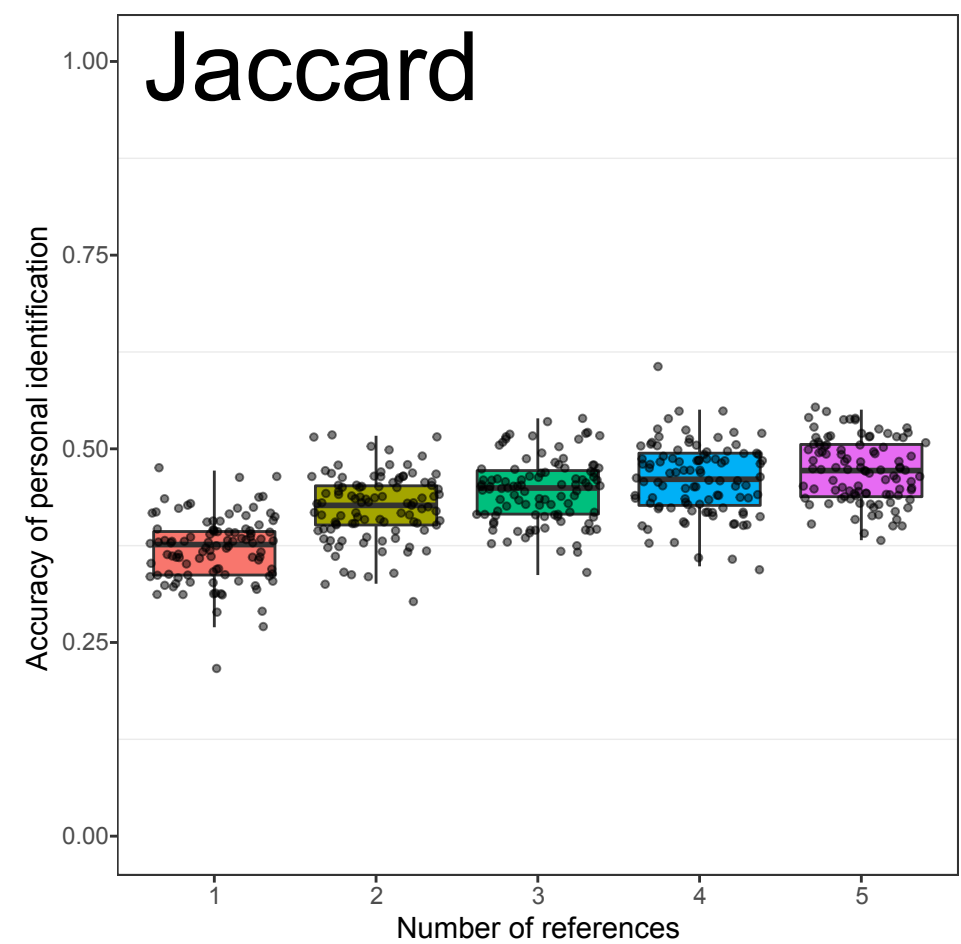

Supplement: S3 Fig — (PDF) [file pone.0199947.s003.pdf]

**A**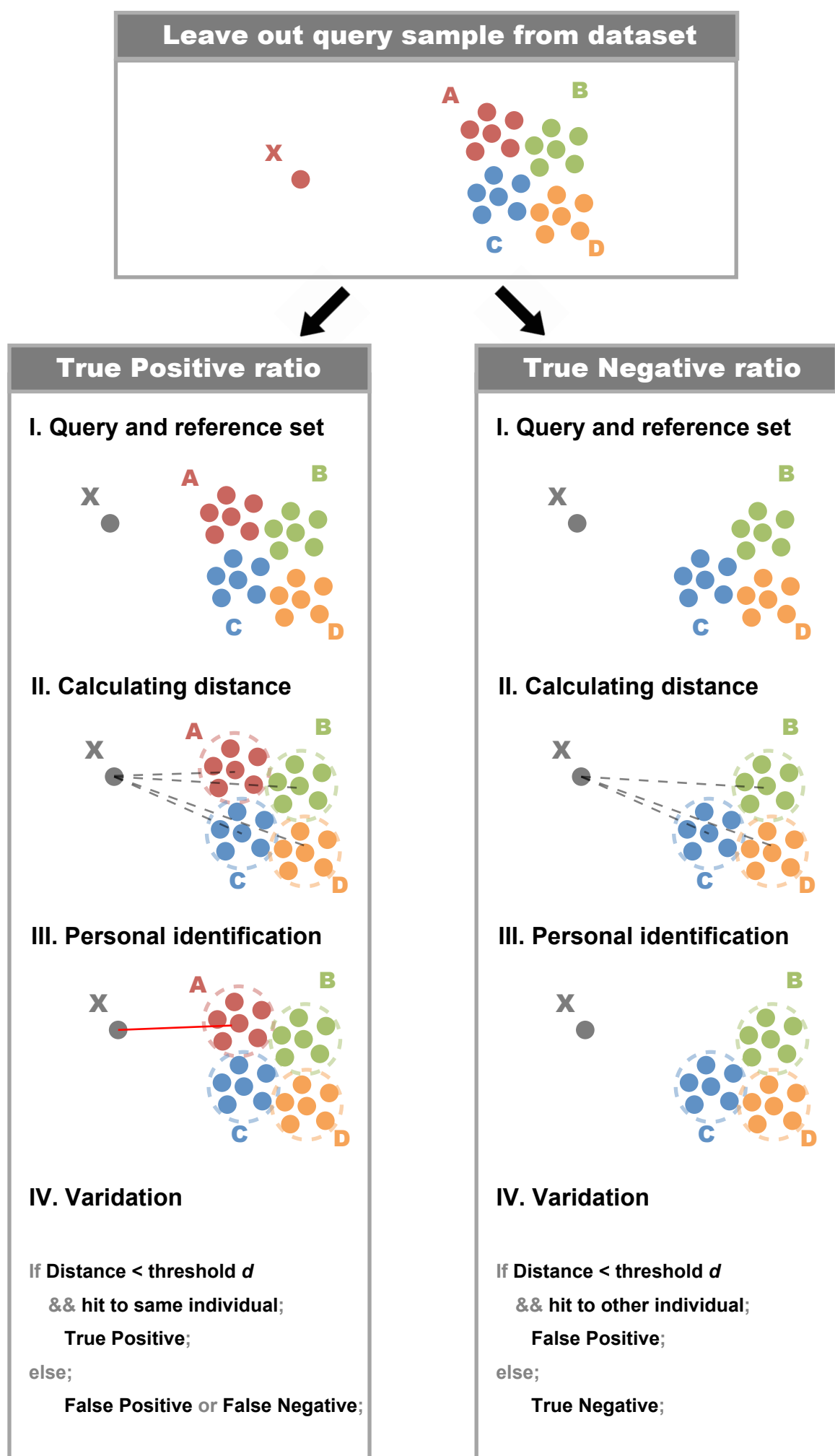**B**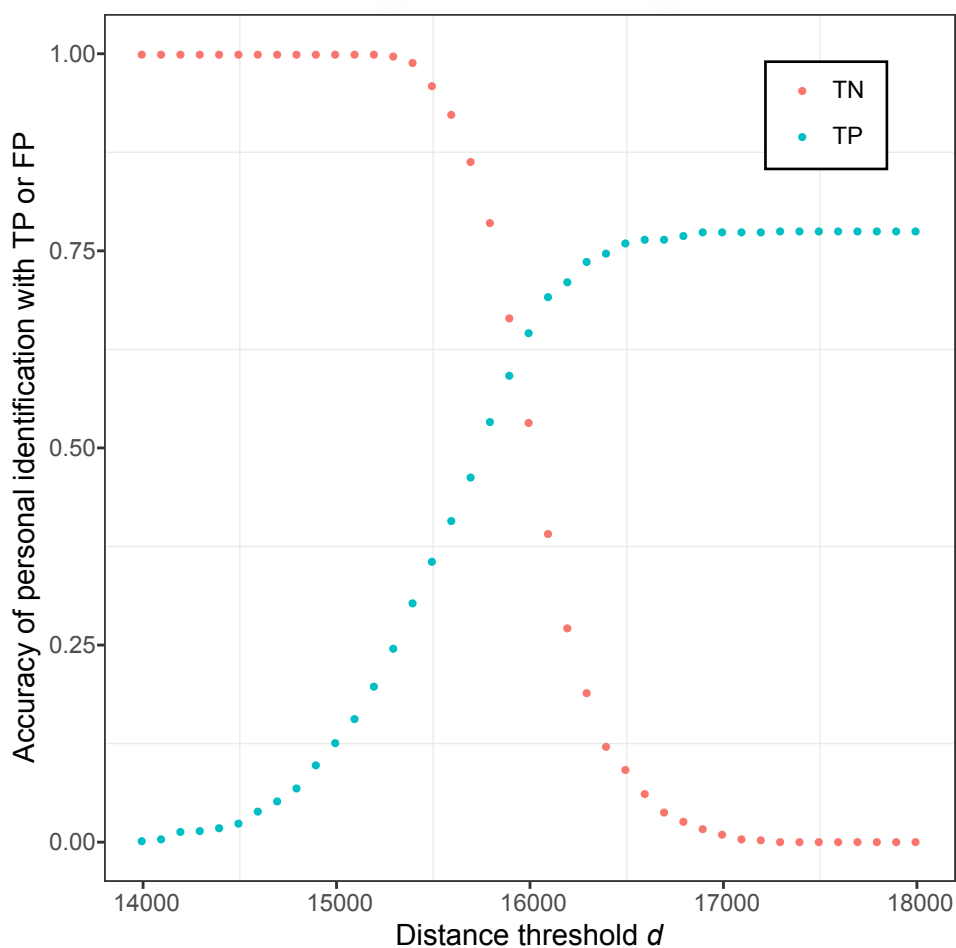

Supplement: S4 Fig — The ratio of TN and TP using our personal identification method by the leave-one-out method. (PDF) [file pone.0199947.s004.pdf]
